# Efficacy and safety evaluations of anlotinib in patients with advanced non-small cell lung cancer treated with bevacizumab

**DOI:** 10.3389/fphar.2022.973448

**Published:** 2022-09-27

**Authors:** Fenge Jiang, Junxia Li, Xiangshuo Kong, Ping Sun, Huajun Qu

**Affiliations:** ^1^ Department of Oncology, The Affiliated Yantai Yuhuangding Hospital of Qingdao University, Yantai, Shandong, China; ^2^ Department of Radiation Oncology, The Affiliated Yantai Yuhuangding Hospital of Qingdao University, Yantai, Shandong, China

**Keywords:** anlotinib, non-small cell lung cancer (NSCLC), bevacizumab, efficacy, safety

## Abstract

**Objective:** The purpose of this study is to evaluate the efficacy and safety of anlotinib in patients with advanced non-small cell lung cancer (NSCLC) who had previously received bevacizumab.

**Methods:** The participants were histopathologically or cytologically diagnosed advanced NSCLC patients whose disease progressed after at least one type of systemic therapy and who had previously received bevacizumab treatment. The patients were on 3-week administration cycles, including 2 weeks on-treatment (12 mg anlotinib oral route, once a day) and 1 week off-treatment. The primary end point of the trial was overall survival (OS). The secondary end points were progression-free survival (PFS), objective response rate (ORR), disease control rate (DCR) and safety.

**Results:** As of the data collection deadline (31 March 2021), 30 patients were enrolled in the study and received anlotinib treatment. All patients were included in the data set except one, who withdrew their consent after the start of treatment. The median follow-up period was 12.1 months (range, 3.6–25.0 months), and 29 patients were included in the evaluation of the treatment. Of the 29 patients, no CR cases occurred. In total, three patients (10.2%) showed a PR, 21 (72.4%) had SD, and five patients (17.2%) had PD. The objective response rate (ORR) was 10.2% (3 of 29 patients), and the disease control rate (DCR) was 82.7% (24 of 29 patients). The median progression-free survival (PFS) was 5.6 months (95% CI, 5.0–6.1 months; Figure 2). The median overall survival (OS) was 10.6 months (95% CI, 9.4–11.8 months; Figure 3). The overall tolerance of the anlotinib treatment was high among the enrolled patients. No treatment-related grade four or five toxicities were observed. Of the 29 patients, one patient’s anlotinib administration was reduced to 8 mg/day due to hypertension and headache. Most adverse events (AEs) were grade one or two; the most common AEs were fatigue (51.7%), hypertension (41.3%), hand–foot syndrome (41.4%), anorexia (34.5%) and hypertriglyceridemia (34.5%).

**Conclusion:** Anlotinib demonstrated favourable activity and manageable toxicity in NSCLC patients who were treated with bevacizumab previously.

## Introduction

Lung cancer is the most commonly diagnosed cancer in the world (22.1% of total cases) and the leading cause of cancer death (36.0% of total cancer deaths) (Siegel et al., 2022). All-cause mortality of lung cancer has become higher than that of breast, prostate, colorectal and brain cancer combined (Siegel et al., 2020). Non-small cell lung cancer (NSCLC) is the most common histological type of lung cancer, which is mainly divided into squamous cell carcinoma and adenocarcinoma, accounting for more than 80% of lung cancer types (Jonna and Subramaniam, 2019). Due to the high invasiveness of NSCLC and the absence of typical symptoms and signs in the early stage, most patients have developed to the middle and late stages at the time of treatment (Duma et al., 2019; Imyanitov et al., 2021). Due to the increase of treatment methods, the survival time of patients with advanced NSCLC has been significantly prolonged, but the 5-years survival rate is still unsatisfactory (Arbour and Riely, 2019). In recent years, many revolutionary advances have been made in the treatment of NSCLC (Folkman, 1971). However, the role of antiangiogenesis therapy in advanced NSCLC is still irreplaceable. The main angiogenic pathways include VEGF, the fibroblast growth factor receptor (FGFR) and the platelet-derived growth factor receptor (PDGFR) (Qiang et al., 2020a). However, bevacizumab inhibits angiogenesis by binding to vascular endothelial growth factor-A (VEGF-A) and inhibiting the vascular endothelial growth factor receptor (VEGFR) signalling pathway, which is conducive to the survival of patients with advanced NSCLC (Reck et al., 2020). Anlotinib is a small molecule multi-target tyrosine kinase inhibitor (TKI), which effectively inhibits VEGFR, PDGFR, FGFR and the stem cell growth factor receptor c-Kit. Based on the findings of the ALTER 0303 trial, anlotinib was approved as a third-line-and-beyond treatment for advanced NSCLC in China as of 9 May 2018 (Jiang et al., 2020). The current clinical indications approved for anlotinib are third-line treatment of advanced NSCLC and first-line treatment of advanced NSCLC. The guidelines also recommend the use of bevacizumab in combination with chemotherapy (Suo et al., 2021). Both bevacizumab and anlotinib are used as antiangiogenic agents; however, the efficacy of retrograde anlotinib remains unclear in patients with advanced NSCLC who were treated with bevacizumab previously. In this study, we investigate the efficacy of anlotinib in patients with advanced NSCLC who had received bevacizumab in previous treatments and investigate the impact of previous antivascular treatment on anlotinib efficacy. This study also provides the basis for the choice of drug in such patients.

## Materials and methods

### Research data

With the consent of the hospital’s Ethics Committee, a single-centre and single-arm study was conducted in Yantai Yuhuangding Hospital, affiliated with Qingdao University, from July 2018 to January 2020. A total of 30 patients were enrolled in this study. One patient withdrew their consent form, and the remaining 29 patients were included in the efficacy and safety analysis and provided written informed consent.

### Inclusion and exclusion criteria

The inclusion criteria were as follows: 1) Aged ≥ 18 years; 2) a histologically confirmed diagnosis of metastatic or recurrent NSCLC; 3) an Eastern Cooperative Oncology Group (ECOG) score of 0 or 1; 4) the patient had at least one measurable lesion; 5) bevacizumab had been used in previous treatments in patients who had received at least one systemic chemotherapy regimen or who could not tolerate treatment. (The patient was usually treated with bevacizumab 15 mg/kg, administered once every 3 weeks. The minimum dosing cycle of bevacizumab in the enrolled patients was one cycle, and the maximum dosing cycle was 19 cycles.); 6) EGFR or ALK positive patients had to undergo targeted therapy for drug resistance or intolerance; 7) normal routine blood tests with liver and kidney functions had to yield ≤ 2.5 and ≤ 1.5 times the normal range, respectively; and 8) life expectancy of more than 12 weeks.

The exclusion criteria were as follows: 1) Small cell lung cancer, including small cell cancer and non-small cell cancer mixed lung cancer; 2) symptomatic brain metastases; and 3) patients whose tumours had invaded important blood vessels or were at risk for massive bleeding during the follow-up studies.

### Research methods

In 3-week cycles, eligible patients were administered 12 mg of anlotinib orally once a day for 14 on-treatment days, followed by 7 off-treatment days, until one of the following circumstances occurred: 1) Disease progression, 2) unacceptable toxicity, 3) withdrawal of consent or 4) death. Anlotinib dosage reductions were given for grade ≥ 3 treatment-related adverse events (TRAEs) or for grade 2 TRAEs lasting more than one cycle. The first anlotinib dose reduction was an adjustment to a/d and the second adjustment was to 8 mg/d. Treatment was terminated if the patients continually showed unacceptable toxicity after the anlotinib reduction.

Relevant medical and study related records were obtained through follow-up methods including telephone communication and the outpatient or inpatient medical records. The follow-up period was until 31 March 2021.

### Evaluating indicator

The primary end point was overall survival (OS). The secondary end points were progression-free survival (PFS), objective response rate (ORR), disease control rate (DCR) and safety. The objective efficacy was evaluated by response evaluation criteria in Solid Tumors version 1.1 (RECIST v1.1) (Eisenhauer et al., 2009). The baseline assessments included the patient’s medical history, physical examination, computed tomography (CT) scan or magnetic resonance imaging and a panel of laboratory tests (e.g.). The efficacy of the treatment was evaluated every 6 weeks (two cycles of anlotinib) by CT scan. The adverse events (AEs) were recorded at baseline and at each visit and were graded according to the National Cancer Institute Common Terminology Criteria for Adverse Events version 5.0 (NCI CTCAE v5.0).

### Statistical methods

All statistical analyses were conducted using SPSS statistical software version 22.0. The proportion of responders was calculated with 95% CIs using the Clopper–Pearson method. The Kaplan-Meier method was used to estimate the median durations of response and PFS with corresponding 95% CIs.

### Follow-up

Relevant medical and study-related records were obtained through follow-up methods, including telephone communication and outpatient or inpatient medical records. The follow-up period was until 31 March 2021.

## Results

### Patient characteristics

The basic characteristics of patients are shown in Table 1. The median age was 57 years (range, 46–72 years). Twenty (69.0%) were male, and nine (31.0%) were female. Thirteen (44%) were never smokers, and 29 (100%) had adenocarcinoma. Four (13.8%) were ECOG 0, and 25 (86.2%) were ECOG 1. Three (10.4%) were Stage IIIB, and 26 (89.6%) were Stage IV. Of 29 patients, 22 (75.9%) received bevacizumab treatment as first-line therapy, 11 (31%) as second-line therapy and three (6.9%) as third-line therapy. Among the 29 patients, four (13.8%) had EGFR mutation and one (3.5%) had ALK fusion mutation. Among them, two (6.9%) were treated with anlotinib as a second-line treatment, 20 (69.0%) as a third-line treatment, and seven (24.1%) as a fourth-line treatment or further-line treatment.

**TABLE 1:** Baseline demographics and disease characteristics.

Characteristic	No. of patients (%) (*N* = 29)	PD (N = 5)	SD (*N* = 21)	PR (*N* = 3)
Age, median (range, yr)	57 (46–72)			
Gender				
Male	20 (69.0%)	3 (10.4%)	15 (51.7%)	2 (6.9%)
Female	9 (31.0%)	2 (6.9%)	6 (20.7%)	1 (3.4%)
Smoking status				
Never	13 (44.8%)	2 (6.9%)	9 (31.0%)	2 (6.9%)
Current	1 (3.4%)	1 (3.4%)	0 (0%)	0 (0%)
Former	15 (51.7%)	2 (6.9%)	12 (41.2%)	1 (3.4%)
ECOG				
0	4 (13.8%)	1 (3.4%)	1 (3.4%)	2 (6.9%)
1	25 (86.2%)	4 (13.8%)	20 (68.7%)	1 (3.4%)
Clinical stage				
IIIB	3 (10.4%)	0 (0%)	2 (6.9%)	1 (3.4%)
IV	26 (89.6%)	5 (17.2%)	19 (65.5%)	2 (6.9%)
Histology type				
Adenocarcinoma	29 (100%)			
Gene mutation				
EGFR mutation	4 (13.8%)			
ALK mutation	1 (3.4%)			
Prior bevacizumab				
1	22 (75.9%)			
2	5 (17.2%)			
3	2 (6.9%)			
Anlotinib				
2	2 (6.9%)	0	1	1
3	20 (69.0%)	2	16	2
4	5 (17.2%)	1	4	0
≥5	2 (6.9%)	2	0	0

ALK, anaplastic lymphoma kinase; ECOG PS, eastern cooperative oncology group performance status; EGFR, epidermal growth factor receptor.

### Efficacy

**FIGURE 1:**
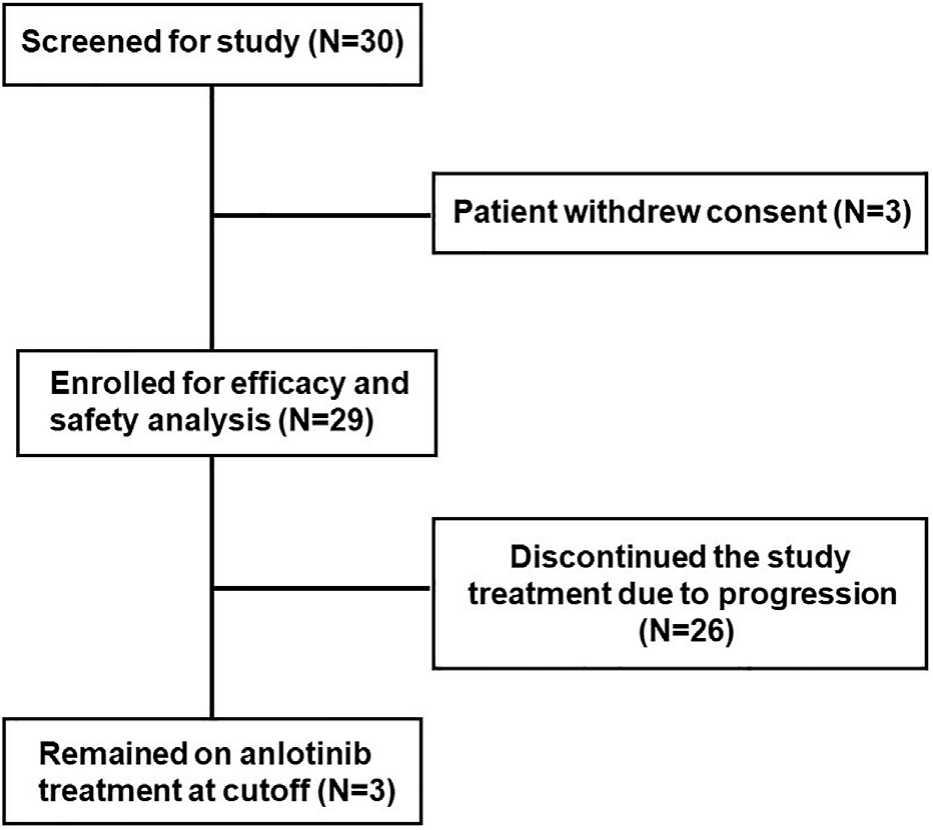
Trial profile, a total of 30 patients were enrolled in this study. One patient withdrew the consent form, and the remaining 29 patients were included in this study.

**FIGURE 2:**
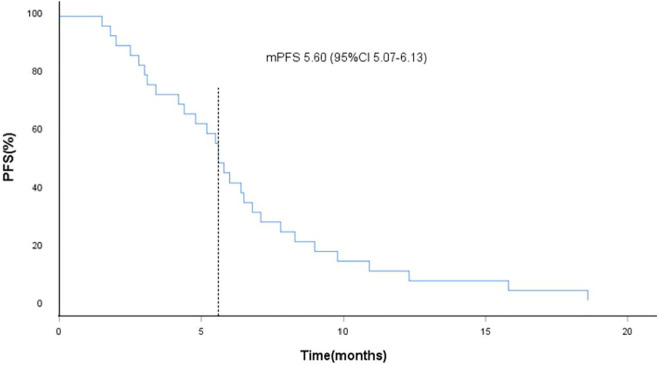
Kaplan-Meier curves of progression-free survival (PFS), the dotted line indicated the median PFS, which is 5.60 months analyzed by Kaplan-Meier method.

**FIGURE 3:**
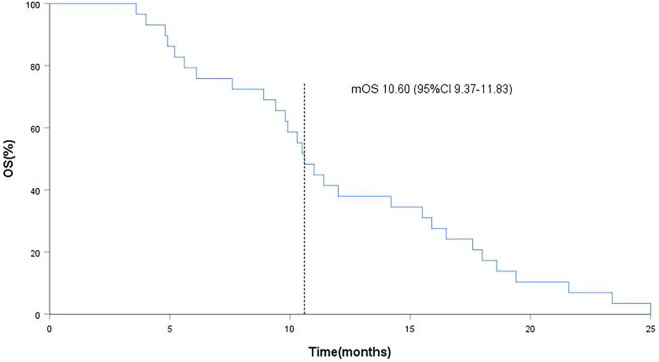
Kaplan-Meier curves of overall survival (OS), the dotted line indicated the median OS, which is 10.60 months analyzed by Kaplan-Meier method.

**FIGURE 4:**
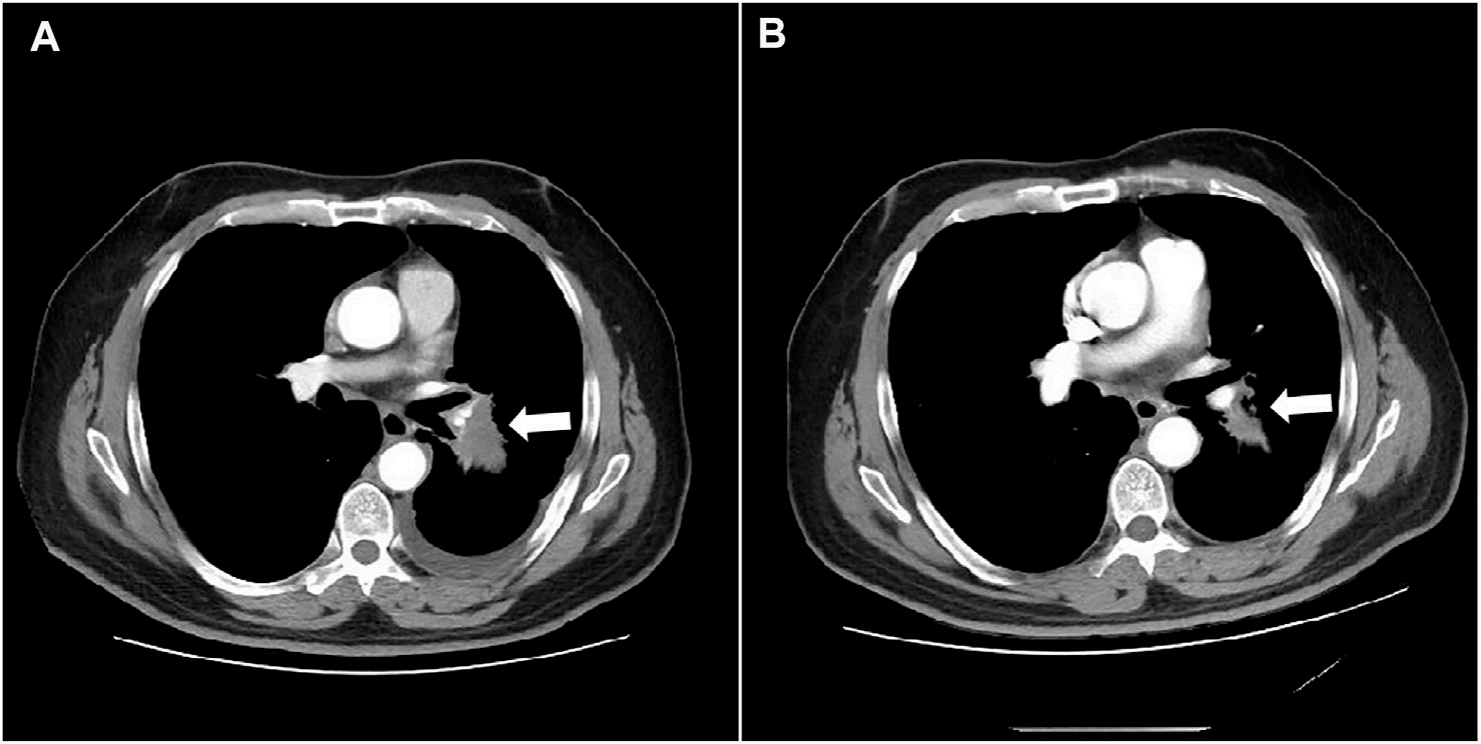
Computed tomography shows the mass in the left lung on mediastinal window before and after treatment **(A)** before anlotinib treatment; **(B)** 4 cycles of anlotinib treatment).

As of data cut-off (31 March 2021), the median follow-up time was 12.1 months (range, 3.6–25.0 months) and 29 patients were included for response evaluation. A total of seven patients are still alive by the primary ending point of this study, of whom three continue to receive anlotinib (Figure 1). No CR case occurred among the 29 participating patients. Three patients (10.2%) showed a PR, 21 (72.4%) had SD, and five patients (17.2%) had PD. The ORR was 10.2% (3 of 29 patients) and the DCR was 82.7% (24 of 29 patients) (Table 2). The number of patients with metastasis and metastasis locations are shown in Table 3. The median PFS was 5.6 months (95% CI, 5.0–6.1 months; Figure 2). The median OS was 10.6 months (95% CI, 9.4–11.8 months; Figure 3). Computer tomography showed a significant reduction of left lung mass in the mediastinal window after four cycles of anlotinib treatment (Figure 4). The size of the tumour was measured, which is shown as 2.6 cm × 1.9 cm in Figure 4A and 1.7fig17 cm × 0.7 cm in Figure 4B.

**TABLE 2:** Confirmed best overall responses.

Response (*N* = 29)	No. (%)
CR	0
PR	3 (10.2%)
SD	21 (72.4%)
PD	5 (17.2%)
ORR	3 (10.2%)
DCR	24 (82.7%)

**TABLE 3:** Demographics of patients with metastasis and metastasis location.

Characteristic	No. of patients (%) (*N* = 29)	PD (*N* = 5)	SD (*N* = 21)	PR (*N* = 3)
Metastase				
Lung metastases	20 (69.0%)	4 (13.8%)	14 (48.3%)	2 (6.9%)
No lung metastases	9 (31.0%)	1 (3.4%)	7 (24.1%)	1 (3.4%)
Liver metastases	9 (31.0%)	3 (10.4%)	6 (20.7%)	0 (0%)
No liver metastases	20 (69.0%)	2 (6.9%)	15 (51.7%)	3 (10.4%)
Bone metastases	11 (37.9%)	2 (6.9%)	9 (31.0%)	0 (0%)
No bone metastase	18 (62.1%)	3 (10.4%)	12 (41.2%)	3 (10.4%)
Brain metastases	5 (17.2%)	2 (6.9%)	3 (10.4%)	0 (0%)
No brain metastases	24 (82.8%)	3 (10.4%)	18 (62.%)	3 (10.4%)

PR, partial response; SD, stable disease; PD, progressive disease.

### Adverse effects

The patients tolerated the treatment well. No treatment-related grade four and grade five toxicities were noted. Of the 29 patients, one patient had a reduction of anlotinib to 8 mg/d due to hypertension and headache. Most AEs were grade 1 or 2, with the most common being fatigue 1 (51.7%), hypertension (41.3%), hand–foot syndrome (41.4%), anorexia (34.5%) and hypertriglyceridemia (34.5%) (Table 4).

**TABLE 4:** Descriptive table of safety shown as adverse events (*n* = 29).

Adverse event	Grade 1–2, N (%)	Grade 3–4, N (%)	All grade, N (%)
Fatigue	15 (51.7%)	0 (0%)	15 (51.7%)
Hypertension	12 (41.3%)	4 (13.8%)	16 (55.2%)
Hand-foot syndrom	11 (38.0%)	1 (3.4%)	11 (38.0%)
Anorexia	10 (34.5%)	0 (0%)	10 (34.5%)
Hypertriglyceridemia	9 (31.0%)	1 (3.4%)	10 (34.5%)
TSH elevation	6 (20.7%)	0 (0%)	6 (20.7%)
Mucositis oral	5 (17.2%)	1 (3.4%)	6 (20.7%)
Proteinuria	4 (13.7%)	0 (0%)	4 (13.7%)
Pharyngalgia	4 (13.7%)	0 (0%)	3 (13.7%)
Diarrhea	4 (13.8%)	0 (0%)	4 (13.8%)
Headache	2 (6.9%)	1 (3.4%)	3 (10.3%)
Hematuria	1 (3.4%)	0 (0%)	1 (3.4%)
hemoptysis	1 (3.4%)	0 (0%)	1 (3.4%)

## Discussion

In recent years, treatments for NSCLC have emerged in an endless stream, but the role of antiangiogenic therapy in NSCLC is still irreplaceable. The concept of antiangiogenesis was proposed by Professor Jodah Folkman in 1971 and has been identified as a potential target for inhibiting tumour progression (Trindade and Duarte, 2020). Since Folkman’s antiangiogenesis theory discovery, many signalling pathways have been identified as key elements in the neoangiogenic process. This has led to the discovery, development and clinical application of antiangiogenic drugs in cancer treatment, such as the anti-VEGF antibody bevacizumab and TKIs, including sorafenib, sunitinib, anlotinib and their analogues. Antiangiogenic agents can act on the tumour microenvironment to degenerate existing tumour blood vessels and inhibit tumour neovascularisation (Qiang et al., 2020b). Antiangiogenic agents have always played an important role in the treatment of advanced NSCLC patients (Folkman, 1972). Tumour growth relies on sufficient blood supply and continuously requires new blood vessel generation, which leads to vascular abnormalities. Antiangiogenic therapy has become a promising treatment regimen in oncology (Falcon et al., 2013; Shibuya, 2013).

Moreover, tumours secrete a variety of pro-angiogenetic factors while growing, and VEGF is the core factor in angiogenesis (Shibuya, 2013). Currently, antiangiogenic agents against the VEGF–VEGFR pathway, including bevacizumab, have shown many benefits in a variety of clinical settings. Bevacizumab exerts the anti-tumour effect mainly in combination with other treatments. Based on the results of a phase III clinical trial, bevacizumab combined with chemotherapy has been approved by the FDA as the standard first-line treatment for non-squamous NSCLC (Hsu and Wakelee, 2009; Patel et al., 2013), and this treatment method has been widely used worldwide. There is currently no standard treatment for advanced NSCLC where bevacizumab has failed.

Anlotinib is a new and effective multi-target TKI, which can play a dual role in inhibiting tumour cell growth and tumour angiogenesis, and combined treatment can improve the drug resistance of chemotherapy drugs (Shen et al., 2018; Gao et al., 2020). Anlotinib has significant inhibitory effects on VEGF-, PDGF-BB- and FGF-2-induced angiogenesis *in vitro* and *in vivo*. Studies have found that anlotinib inhibits VEGF-, PDGF-BB-, and FGF-2-induced cell migration and formation of capillary-like tubes in endothelial cells. According to research, the antiangiogenic effect of anlotinib is superior to sunitinib, sorafenib and nintedanib, which are the three main clinically used antiangiogenesis drugs (Lin et al., 2018). Mechanistically, anlotinib inhibits the activation of VEGFR2, PDGFRb, and FGFR1 as well as their common downstream ERK signalling (Lin et al., 2018). This prospective study demonstrates the efficacy of anlotinib in advanced NSCLC patients who were previously treated with bevacizumab.

In this study, the OS and PFS were 10.6 and 5.6 months, respectively, which were slightly higher than the 9.6 and 5.4 months reported in the ALTER 0303 study. The possible reasons could be the following. First, most of the people enrolled in our study were in third-line treatment and some were in second-line treatment, whereas the patients enrolled in the ALTER 0303 study were involved in third-line treatments or beyond. This discrepancy may also be related to the small sample size of this study, of which some patients with a high ECOG PS score were enrolled. The ALTER 0303 trial subgroup analysis (Wang et al., 2019) showed that there was no statistical difference in PFS and OS among patients who had previously received bevacizumab or endostatin or had never received either treatment. However, only 11 patients in the subgroup had been previously treated with bevacizumab, and the sample size was small. This study further confirms that previous bevacizumab treatment did not affect the efficacy of anlotinib. The main AEs in this study were fatigue (51.7%), hypertension (41.4%), hand–foot syndrome (38.0%), anorexia (34.5%) and hypertriglyceridemia (34.5%). Most adverse events were mild and could be managed by symptomatic relief treatment or dosage adjustment. There was no increased risk of bleeding in these patients, including the ones previously treated with bevacizumab. Haematuria and haemoptysis occurred in only one of the 29 patients, all with grade 1 AEs. One 60-year-old female patient, who denied a history of hypertension in the past, was treated with oral anlotinib and developed grade 3 hypertension on day 10. At the same time, the patient experienced headache and intolerance. Because of this, the per-day dose was reduced to 8 mg of anlotinib and oral administration of 30 mg nifedipine controlled-release tablets. Their blood pressure could be controlled within the normal range, their headache symptoms disappeared and the patient did not affect the curative effect. The re-examination reached PR after four cycles (Figure 4).

## Conclusion

In summary, anlotinib showed good activity and safety in NSCLC patients treated with bevacizumab in the past. The clinical therapeutic effect was remarkable, which could effectively prolong the OS and PFS of patients. Anlotinib has certain clinical value for NSCLC patients treated with bevacizumab, which may provide new treatment options for advanced NSCLC patients treated with bevacizumab. However, due to the single centre and small sample size of this study, there may be a certain selection bias. In the future, a large number of multicentre and randomised controlled trials are needed to further evaluate the efficacy and safety of anlotinib in the treatment of advanced NSCLC previously treated with bevacizumab.

## Data Availability

The original contributions presented in the study are included in the article/supplementary material, further inquiries can be directed to the corresponding authors.

## References

[B1] Arbour K. C. Riely G. J. (2019). Systemic therapy for locally advanced and metastatic non-small cell lung cancer: A review. JAMA 322 (8), 764–774. 10.1001/jama.2019.11058 31454018

[B2] Duma N. Santana-Davila R. Molina J. R. (2019). Non-small cell lung cancer: Epidemiology, screening, diagnosis, and treatment. Mayo Clin. Proc. 94 (8), 1623–1640. 10.1016/j.mayocp.2019.01.013 31378236

[B3] Eisenhauer E. A. Therasse P. Bogaerts J. Schwartz L. H. Sargent D. FoRd R. (2009). New response evaluation criteria in solid tumours: Revised RECIST guideline (version 1.1). Eur. J. Cancer 45 (2), 228–247. 10.1016/j.ejca.2008.10.026 19097774

[B4] Falcon B. L. O'Clair B. Swearingen M. L. Evans G. F. Stewart J. Chen Y. (2013). Development and characterization of a high-throughput *in vitro* cord formation model insensitive to VEGF inhibition. J. Hematol. Oncol. 6, 31. 10.1186/1756-8722-6-31 23622716 PMC3648446

[B5] Folkman J. (1971). Tumor angiogenesis: Therapeutic implications. N. Engl. J. Med. 285, 1182–1186. 10.1056/NEJM197111182852108 4938153

[B6] Folkman J. (1972). Anti-angiogenesis: New concept for therapy of solid tumors. Ann. Surg. 175 (3), 409–416. 10.1097/00000658-197203000-00014 5077799 PMC1355186

[B7] Gao Y. Liu P. Shi R. (2020). Anlotinib as a molecular targeted therapy for tumors. Oncol. Lett. 20 (2), 1001–1014. 10.3892/ol.2020.11685 32724339 PMC7377159

[B8] Hsu J. Y. Wakelee H. A. (2009). Monoclonal antibodies targeting vascular endothelial growth factor: Current status and future challenges in cancer therapy. BioDrugs 23, 289–304. 10.2165/11317600-000000000-00000 19754219

[B9] Imyanitov E. N. Iyevleva A. G. Levchenko E. V. (2021). Molecular testing and targeted therapy for non-small cell lung cancer: Current status and perspectives. Crit. Rev. Oncol. Hematol. 157, 103194. 10.1016/j.critrevonc.2020.103194 33316418

[B10] Jiang S. Liang H. Liu Z. Zhao S. Liu J. Xie Z. (2020). The impact of anlotinib on brain metastases of non-small cell lung cancer: Post hoc analysis of a phase III randomized control trial (ALTER0303). Oncologist 25 (5), e870–e874. 10.1634/theoncologist.2019-0838 32077550 PMC7216446

[B11] Jonna S. Subramaniam D. S. (2019). Molecular diagnostics and targeted therapies in non-small cell lung cancer (NSCLC): An update. Discov. Med. 27 (148), 167–170. 31095926

[B12] Lin B. Song X. Lu N. Bai D. Yao Y. (2018). Anlotinib inhibits angiogenesis via suppressing the activation of VEGFR2, PDGFRβ and FGFR1. Gene 654, 77–86. 10.1016/j.gene.2018.02.026 29454091

[B13] Patel J. D. Socinski M. A. Garon E. B. Reynolds C. H. Spigel D. R. Olsen M. R. (2013). PointBreak: A randomized phase III study of pemetrexed plus carboplatin and bevacizumab followed by maintenance pemetrexed and bevacizumab versus paclitaxel plus carboplatin and bevacizumab followed by maintenance bevacizumab in patients with stage IIIB or IV nonsquamous non-small-cell lung cancer. J. Clin. Oncol. 31, 4349–4357. 10.1200/JCO.2012.47.9626 24145346 PMC4881367

[B14] Qiang H. Chang Q. Chu T. Qian J. Zhang Y. Lei Y. (2020). New advances in antiangiogenic combination therapeutic strategies for advanced non-small cell lung cancer. J. Cancer Res. Clin. Oncol. 146 (3), 631–645. 10.1007/s00432-020-03129-6 32065262 PMC11804424

[B15] Qiang H. Chang Q. Chu T. Qian J. Zhang Y. Lei Y. (2020). New advances in antiangiogenic combination therapeutic strategies for advanced non-small cell lung cancer. J. Cancer Res. Clin. Oncol. 146 (3), 631–645. 10.1007/s00432-020-03129-6 32065262 PMC11804424

[B16] Reck M. Shankar G. Sandler A. Coleman S. McCleland M. Papadimitrakopoulou V. A. (2020). Atezolizumab in combination with bevacizumab, paclitaxel and carboplatin for the first-line treatment of patients with metastatic non-squamous non-small cell lung cancer, including patients with EGFR mutations. Expert Rev. Respir. Med. 14 (2), 125–136. 10.1080/17476348.2020.1701439 31829747

[B17] Shen G. Zheng F. Zhao J. Du F. Dong Q. Wang Z. (2018). Anlotinib: A novel multi-targeting tyrosine kinase inhibitor in clinical development. J. Hematol. Oncol. 11 (1), 120. 10.1186/s13045-018-0664-7 30231931 PMC6146601

[B18] Shibuya M. (2013). VEGFR and type-V RTK activation and signaling. Cold Spring Harb. Perspect. Biol. 5, a009092. 10.1101/cshperspect.a009092 24086040 PMC3783052

[B19] Siegel R. L. Miller K. D. Jemal A. (2020). Cancer statistics, 2020. Ca. Cancer J. Clin. 70 (1), 7–30. 10.3322/caac.21590 31912902

[B20] Siegel R. L. Miller K. D. Fuchs H. E. Jemal A. (2022). Cancer statistics, 2022. Ca. Cancer J. Clin. 72 (1), 7–33. 10.3322/caac.21708 35020204

[B21] Suo J. Sun Y. Fu Y. Xiu W. Zhang X. Wang Y. (2021). A retrospective analysis of the effect of anlotinib in patients with lung cancer with or without previous antiangiogenic therapy. Front. Oncol. 11, 788837. 10.3389/fonc.2021.788837 35004313 PMC8732369

[B22] Trindade A. Duarte A. (2020). Notch signaling function in the angiocrine regulation of tumor development. Cells 9 (11), 2467. 10.3390/cells9112467 33198378 PMC7697556

[B23] Wang L. He Z. Wang Q. Tang H. Wu Y. Li S. (2019). The impact of previous therapy strategy on the efficiency of anlotinib hydrochloride as a third-line treatment on patients with advanced non-small cell lung cancer (NSCLC): A subgroup analysis of ALTER0303 trial. Transl. Lung Cancer Res. 8 (5), 575–583. 10.21037/tlcr.2019.09.21 31737494 PMC6835105

