# Erratum: Efficacy and safety evaluations of anlotinib in patients with advanced non-small cell lung cancer treated with bevacizumab

**DOI:** 10.3389/fphar.2022.1092147

**Published:** 2022-11-22

**Authors:** Frontiers Production Office

**Affiliations:** Frontiers Media SA, Lausanne, Switzerland

**Keywords:** anlotinib, non-small cell lung cancer (NSCLC), bevacizumab, efficacy, safety

Due to a mistake on the publisher’s part, this article was originally published as a Systematic Review instead of Original Research.

The publisher apologizes for this error and states that this does not change the scientific conclusions of the article in any way. The original article has been updated.

